# Multiple gene promoter methylation and clinical stage in adjacent normal tissues: Effect on prognosis of colorectal cancer in Taiwan

**DOI:** 10.1038/s41598-019-56691-6

**Published:** 2020-01-10

**Authors:** Chih-Hsiung Hsu, Cheng-Wen Hsiao, Chien-An Sun, Wen-Chih Wu, Tsan Yang, Je-Ming Hu, Yu-Chan Liao, Chi-Hua Huang, Chao-Yang Chen, Fu-Huang Lin, Yu-Ching Chou

**Affiliations:** 1 Graduate Institute of Medical Sciences, National Defense Medical Center, Taipei, Taiwan, Republic of China; 2 Teaching Office, Tri-Service General Hospital, National Defense Medical Center, Taipei, Taiwan, Republic of China; 3 Division of Colorectal Surgery, Department of Surgery, Tri-Service General Hospital, National Defense Medical Center, Taipei, Taiwan, Republic of China; 4 Department of Public Health, College of Medicine, Fu Jen Catholic University, New Taipei City, Taiwan, Republic of China; 5 Big Data Research Center, College of Medicine, Fu Jen Catholic University, New Taipei City, Taiwan, Republic of China; 6 School of Public Health, National Defense Medical Center, Taipei, Taiwan, Republic of China; 7 Department of Surgery, Suao and Yuanshan branches of Taipei Veterans General Hospital, Yilan County, Taiwan, Republic of China; 8 Department of Health Business Administration, Meiho University, Pingtung County, Taiwan, Republic of China; 9 Adjunct Instructor, School of Medicine, National Defense Medical Center, Taipei, Taiwan, Republic of China

**Keywords:** Prognostic markers, Cancer epidemiology

## Abstract

This study provide an insight that the panel genes methylation status in different clinical stage tended to reflect a different prognosis even in matched normal tissues, to clinical recommendation. We enrolled 153 colorectal cancer patients from a medical center in Taiwan and used the candidate gene approach to select five genes involved in carcinogenesis pathways. We analyzed the relationship between DNA methylation with different cancer stages and the prognostic outcome. There were significant trends of increasing risk of 5-year time to progression and event-free survival of subjects with raising number of hypermethylation genes both in normal tissue and tumor tissue. The group with two or more genes with aberrant methylation in the advanced cancer stages (Me/advanced) had lower 5-year event-free survival among patients with colorectal cancer in either normal or tumor tissue. The adjusted hazard ratios in the group with two or more genes with aberrant methylation with advanced cancer stages (Me/advanced) were 8.04 (95% CI, 2.80–23.1; P for trend <0.01) and 8.01 (95% CI, 1.92–33.4; P for trend <0.01) in normal and tumor tissue, respectively. DNA methylation status was significantly associated with poor prognosis outcome. This finding in the matched normal tissues of colorectal cancer patients could be an alternative source of prognostic markers to assist clinical decision making.

## Introduction

Colorectal cancer (CRC) is the second most frequently diagnosed cancer in women (614,000 cases, 9.2% of the total female population) and the third most frequently diagnosed cancer in men (746,000 cases, 10% of the total male population) worldwide^1–3^. The prognosis of and options of therapy for CRC rely on pathological stages, the staging system used (Dukes system or the TNM system), 5-year survival rate, and disease-free rate. It is fabulous for early-stage disease, ranging between 91% and 80% for histological stages I and II, in patients who can benefit from curative treatment, but it is poor in patients with isolated liver or lung metastases (5-year survival ≤20%)^4–7^. Over the past two decades, various treatment strategies have resulted in significant improvement in survival among patients with CRC^8^. Extensive studies have been executed to recognize novel prognostic and predictive biomarkers for CRC, including both genetic and epigenetic abnormalities. Previous studies have demonstrated that aberrant DNA methylation, the most frequent aberrant epigenetic modification in cancer, is an important early biomarker in CRC that is related to transcriptional silencing of tumor suppressor genes and a marker for field cancerization^9–11^.

With these biomarkers, patients in the same tumor stage could be stratified by different individual molecular factors. It’s useful for prognosis prediction and individualized treatment^12,13^. Previous studies have identified that methylation of cyclin-dependent kinase inhibitor 2A (*CDKN2A*), O-6-methylguanine DNA methyltransferase (*MGMT*) and human mutL homolog 1 (*hMLH1*), correlated with carcinogenesis pathways through gene silencing, could serve as diagnostic prognostic markers for CRC^14–17^. We selected two other candidate genes, colony stimulating factor 2 (*CSF2*) and DIS3 mitotic control homolog (*S. cerevisiae*)-like 2 (*DIS3L2*), from a previous study database^18^ that involved inhibitory effects on tumor growth^19–22^. To understand the effect of specific gene methylation on the relationship between CRC prognosis including progression and mortality and histological stage, we explored DNA methylation status in tumor and adjacent normal tissues (matched normal) from subjects who received surgical resection for CRC. In this study, we provide insight that the selected genes methylation status in different clinical stages tended to reflect a different prognosis, even in matched normal tissues, to clinical recommendations.

## Methods

### Patients and specimen collections

We designed a hospital-based retrospective cohort study to estimate the 5-year prognosis of patients with CRC in Taiwan and have been described elsewhere^17,23^. Patients with a diagnosis of invasive CRC between 2006 and 2010 who underwent surgical resection were eligible for recruitment. Tumor stage was defined according to the American Joint Committee on Cancer TNM staging system^24^. Informed consent was obtained from all enrollees to evaluate prognosis (including recurrence, metastasis, and survival). This study was approved by the Institutional Review Board (IRB) of Tri-Service General Hospital (TSGH) (certificated number 098-05-292 and 2-105-05-129). The method of obtaining follow-up data for registered patients, including patients’ information on the prognosis including recurrence, metastasis and the cause of death, relied on medical records linked to data in a cancer registration database. In this study, according to the clinical practice guideline of the TSGH Division of Colon and Rectum, the enrollees should return for a checkup once every 3 months in the first year after receiving surgical resection and once every 3–6 months subsequently. All methods were organized under, and operated per International Conference on Harmonization (ICH) / WHO GCP and the applicable laws and regulations. In addition, we also confirmed that all methods were performed in accordance with the relevant guidelines and regulations. Progression was defined as local recurrence or metastases. Time to progression (TTP) has been described as the time interval from the date of receiving surgical resection to the date of disease progression. Overall survival (OS) was calculated from the date of receiving surgical resection to the date of death of any cause. Event-free survival (EFS) was defined as the interval from the date of receiving surgical resection to the date of disease progression or death as a result of any cause. Otherwise, the enrollees without progression and who were survivors were followed up until the latest date of checkup as the study endpoint.

**Figure 1:**
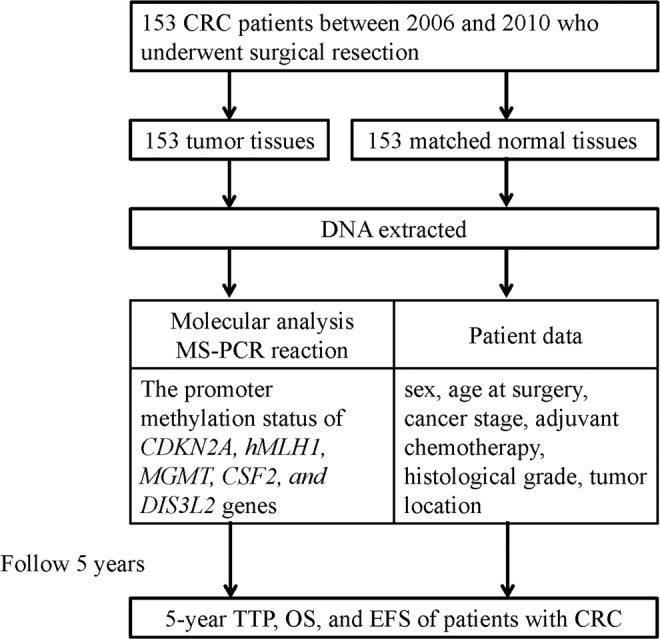
Flowchart of study design.

Data on sex, age at surgery, cancer stage, adjuvant chemotherapy, histological grade, and tumor location were obtained from the patients’ medical files. On the basis of the inclusion criteria, we identified 153 tumor tissues and matched normal tissues (306 samples) from enrollees in TSGH. The mean values for TTP, OS, and EFS were 2.67 years, 3.53 years, and 3.53 years, respectively. The fresh tissue samples of participants were obtained in the operating room while tumor tissues were resected. From each patient, adjacent normal mucosa tissue samples were collected from resected, unaffected parts of the colon located at least 10 cm from the tumor site. Samples were immediately frozen in liquid nitrogen and stored at −80 °C for subsequent DNA extraction and methylation assays. Figure 1 depicts the flowchart of the study design.

### DNA extracted and MS-PCR reaction

We used the Genomic DNA Tissue Kit (catalogue no. 69504; Qiagen, Taipei, Taiwan) to extract the genomic DNA from the tissues according to the manufacturer’s protocol and the cellulose-coated magnetic beads with the MagCore Compact Automated Nucleic Acid Extractor (catalogue no. MCA0801; RBC Bioscience, Taipei, Taiwan). The isolated DNA was treated with sodium bisulfite using the EZ DNA Methylation Kit (Zymo Research Corporation, Orange, CA, USA).

We evaluated the promoter methylation status of *CDKN2A*, *hMLH1*, *MGMT*, *CSF2*, and *DIS3L2* genes through methylation-specific polymerase chain reaction (MS-PCR), as described in our previous research^17,23^. The reaction solution (25 μL) contained 1.2-μL aliquots of forward and reverse primers, 12.5 μL HotStart Taq Premix (RBC Bioscience) and bisulfite-converted DNA. The sequences, annealing temperature of each primer used for amplification, and PCR product sizes are described in Table 1. PCR cycling conditions were as follows: first, 10 min at 95 °C; then, 35 cycles of 30-s denaturation at 95 °C, 30-s annealing, and 30-s extension at 72 °C; finally, 4-min extension at 72 °C. After the amplification, PCR products were mixed with a loading buffer, electrophoresed on 2% agarose gel by using 0.2-μL gel-stained dye for 25 min, and visualized using an ultraviolet transilluminator.

**Table 1:** Primer sequences, annealing temperature and product size for MSP of target genes.

Genes		Forward primer (5′ → 3′)	Annealing temperature(oC)	Product size (bp)
*CDKN2A*	M	F:TTATTAGAGGGTGGGGCGGATCGC	62	150
		R:GACCCCGAACCGCGACCGTAA		
	U	F:TTATTAGAGGGTGGGGTGGATTGT	62	151
		R:CAACCCCAAACCACAACCATAA		
*hMLH1*	M	F:ACGTAGACGTTTTATTAGGGTCGC	60	118
		R:CCTCATCGTAACTACCCGCG		
	U	F:TTTTGATGTAGATGTTTTATTAGGGTTGT	60	124
		R:ACCACCTCATCATAACTACCCACA		
*MGMT*	M	F:TTTCGACGTTCGTAGGTTTTCGC	53	81
		R:GCACTCTTCCGAAAACGAAACG		
	U	F:TTTGTGTTTTGATGTTTGTAGGTTTTTGT	53	93
		R:AACTCCACACTCTTCCAAAAACAAAACA		
*CSF2*	M	F: TGATTATTTAGGGAAAAGGTTTATC	56	105
		R: ATAACCACAAAATACCAAAAAAACG		
	U	F: ATTATTTAGGGAAAAGGTTTATTGT	60	104
		R: AATAACCACAAAATACCAAAAAAACA		
*DIS3L2*	M	F: GTCGTAGTTGAATCGTCGATTAC	54	134
		R: TTACTAAAAAAAATACTCTTCCGAA		
	U	F: GTTGTAGTTGAATTGTTGATTATGA	55	134
		R: TTACTAAAAAAAATACTCTTCCAAA		

MSP, methylation-specific polymerase chain reaction; CDKN2A, cyclin-dependent kinase inhibitor 2A; MLH1, mutL homolog 1; MGMT, O-6-methylguanine DNA methyltransferase; CSF2, colony stimulating factor 2; DIS3L2, DIS3 mitotic control homolog (S. cerevisiae)-like 2; M, methylation; U, unmethylation.

### Statistical analysis

To determine the association of methylation in *CDKN2A*, *hMLH1*, *MGMT*, *CSF2*, and *DIS3L2* with 5-year TTP, OS, and EFS of patients with CRC in different clinical stages, we separately evaluated the various stages and divided them into two subgroups (local and advanced stages) on the basis of the different pathological types of tissue: tumor and adjacent normal tissues (matched normal).

Patients have been split into two groups based on the methylation status of the five evaluated genes: a group with aberrant methylation at two or more genes and a group with aberrant methylation at less than two genes. We used the Kaplan–Meier survival analysis to estimate 5-year TTP, OS, and EFS. Log-rank tests were used to assess the significance of differences in the groups. We performed multivariate analyses with a Cox proportional hazards model adjusted for baseline characteristics, which included sex, age at surgery (continuous), cancer stage (1, 2, 3, or 4), adjuvant chemotherapy, lymphovascular invasion, histological grade, tumor location, and methylation status of candidate genes based on previous studies^23^ to evaluate hazard ratios (HRs) with 95% confidence intervals (CIs). All statistical tests were two-sided, and *P* values < 0.05 were considered statistically significant. Statistical analyses for the clinical study were conducted using IBM SPSS Statistics version 22 (IBM® SPSS® Statistics 22).

## Results

During the study period, we identified 153 tumor samples of patients with CRC and matched normal samples from the TSGH tumor bank. The demographic features of the study population are shown in Table 2. Among the study patients, 51.0% were men, and the mean age was 64.1 years (standard deviation, 14.7 years). The patients were classified into four clinical subgroups: stage 1 (15.0%), stage 2 (35.3%), stage 3 (32.0%), and stage 4 (17.6%). The progression in 5 years indicated that 41.2% of the enrollees had cancer recurrence or metastasis and 38.5% died. In addition, on the basis of the tumor and matched normal tissues of the patients, we classified the patient characteristics according to five individual gene groups (*CDKN2A*, *hMLH1*, *MGMT*, *CSF2*, and *DIS3L2*) and according to those with two or more genes with aberrant methylation, stratified by different variables (sex, age at surgery, cancer stage, progression, all-cause death, progression including death, adjuvant chemotherapy, histological grade, and tumor location).

**Table 2:** Characteristics and distribution of methylation status in patients with CRC (n = 153).

Variables	*Total*	Methylation status					
		*CSF2*		* DIS3L2*		≥2 of genes	
		Normal	Tumors	Normal	Tumors	Normal	Tumors
**Sex, n (%)**							
Male	78 (51.0)	21 (26.9)	49 (62.8)	18 (23.1)	19 (24.4)	17 (21.8)	47 (60.3)
Female	75 (49.0)	28 (37.3)	43 (57.3)	17 (22.7)	20 (26.7)	10 (13.3)	39 (52.0)
**Age at surgery**							
Mean (SD)	64.1 (14.7)	63.1 (15.0)	62.6 (16.3)	66.7 (12.6)	68.1 (13.1)	61.3 (16.0)	63.3 (14.8)
<65, n (%)	25 (33.3)	51 (68.0)	59 (77.6)	15 (20.0)	16 (21.3)	16 (21.3)	45 (60.0)
≥65, n (%)	24 (30.8)	41 (52.6)	58 (74.4)	20 (25.6)	23 (29.5)	11 (14.1)	41 (52.6)
**Stage, n (%)**							
I	23 (15.0)	9 (39.1)	11 (47.8)	3 (13.0)	6 (26.1)	1 (4.3)	8 (34.8)
II	54 (35.3)	19 (35.2)	34 (63.0)	12 (22.2)	10 (18.5)	9 (16.7)	30 (55.6)
III	49 (32.0)	15 (30.6)	31 (63.3)	16 (32.7)	15 (30.6)	8 (16.3)	33 (67.3)
IV	27 (17.6)	6 (22.2)	16 (59.3)	4 (14.8)	8 (29.6)	9 (33.3)	15 (55.6)
**Progression in 5** **yr, n (%)**							
No	90 (58.8)	26 (28.9)	52 (57.8)	17 (18.9)	20 (22.2)	10 (11.1)	43 (47.8)
Yes	63 (41.2)	23 (36.5)	40 (63.5)	18 (28.6)	19 (30.2)	17 (27.0)	43 (68.3)
**All-cause death in 5** **yr, n (%)**							
No	122 (79.7)	39 (32.0)	76 (62.3)	30 (24.6)	33 (27.0)	20 (16.4)	71 (58.2)
Yes	31 (20.3)	10 (32.3)	16 (51.6)	5 (16.1)	6 (193.4)	7 (22.6)	15 (48.4)
**Progression including death, in 5** **yr, n (%)**							
No	78 (51.0)	22 (28.2)	44 (56.4)	15 (19.2)	17 (21.8)	7 (9.0)	37 (47.4)
Yes	75 (49.0)	27 (36.0)	48 (64.0)	20 (26.7)	22 (29.3)	20 (26.7)	49 (65.3)
**Adjuvant chemotherapy, n (%)**							
No	37 (24.2)	20 (54.1)	28 (75.7)	16 (43.2)	16 (43.2)	6 (16.2)	21 (56.8)
Yes	97 (63.4)	26 (26.8)	57 (58.8)	17 (17.5)	20 (20.6)	20 (20.6)	57 (58.8)
Histological grade, n (%)*							
Well or Moderately	114 (74.6)	39 (34.2)	69 (60.5)	29 (25.4)	31 (27.2)	23 (20.2)	62 (54.4)
Poor or undifferentiated	13 (8.5)	2 (15.4)	9 (69.2)	1 (7.7)	3 (23.1)	3 (23.1)	10 (76.9)
Tumor location, n (%)*							
Colon	104 (68.0)	32 (30.8)	63 (60.6)	25 (24.0)	25 (24.0)	20 (19.2)	58 (55.8)
Rectum	30 (19.6)	14 (46.7)	22 (73.3)	8 (26.7)	11 (36.7)	6 (20.0)	20 (66.7)
**Variables**	***Total***	**Methylation status**					
		***CSF2***		***DIS3L2***		**≥2 of genes**	
		**Normal**	**Normal**	**Normal**	**Tumors**	**Normal**	**Tumors**
**Sex, n (%)**							
Male	78 (51.0)	51 (65.4)	57 (73.1)	21 (26.9)	26 (33.3)	41 (52.6)	62 (79.5)
Female	75 (49.0)	61 (81.3)	60 (80.0)	24 (32.0)	28 (37.3)	45 (60.0)	63 (84.0)
**Age at surgery**							
Mean (SD)	64.1 (14.7)	62.4 (14.9)	63.3 (14.9)	59.7 (14.7)	61.8 (15.0)	61.3 (14.6)	63.3 (15.3)
<65, n (%)	25 (33.3)	61 (81.3)	59 (78.7)	28 (37.3)	33 (44.0)	49 (65.3)	65 (86.7)
≥65, n (%)	24 (30.8)	51 (65.4)	58 (74.4)	17 (21.8)	21 (26.9)	37 (47.4)	60 (76.9)
**Stage, n (%)**							
I	23 (15.0)	17 (73.9)	19 (82.6)	9 (39.1)	7 (30.4)	13 (56.5)	15 (65.2)
II	54 (35.3)	39 (72.2)	39 (72.2)	15 (27.8)	19 (35.2)	28 (51.9)	42 (77.8)
III	49 (32.0)	35 (71.4)	37 (75.5)	11 (22.4)	14 (28.6)	28 (57.1)	45 (91.8)
IV	27 (17.6)	21 (77.8)	22 (81.5)	10 (37.0)	14 (51.9)	17 (63.0)	23 (85.2)
**Progression in 5** **yr, n (%)**							
No	90 (58.8)	65 (72.2)	67 (74.4)	27 (30.0)	32 (35.6)	45 (50.0)	69 (76.7)
Yes	63 (41.2)	47 (74.6)	50 (79.4)	18 (28.6)	22 (34.9)	41 (65.1)	56 (88.9)
**All-cause death in 5** **yr, n (%)**							
No	122 (79.7)	86 (70.5)	89 (73.0)	37 (30.3)	41 (33.6)	69 (56.6)	100 (82.0)
Yes	31 (20.3)	26 (83.9)	28 (90.3)	8 (25.8)	13 (41.9)	17 (54.8)	25 (80.6)
**Progression including death, in 5** **yr, n (%)**							
No	78 (51.0)	53 (67.9)	55 (70.5)	25 (32.1)	28 (35.9)	38 (48.7)	58 (74.4)
Yes	75 (49.0)	59 (78.7)	82 (82.7)	20 (26.7)	26 (34.7)	48 (64.0)	67 (89.3)
**Adjuvant chemotherapy, n (%)**							
No	37 (24.2)	30 (81.1)	34 (91.9)	14 (37.8)	14 (37.8)	29 (78.4)	34 (91.9)
Yes	97 (63.4)	71 (73.2)	72 (74.2)	29 (29.9)	39 (40.2)	51 (52.6)	81 (83.5)
Histological grade, n (%)*							
Well or Moderately	114 (74.6)	83 (72.8)	87 (76.3)	36 (31.6)	45 (39.5)	67 (58.8)	95 (83.3)
Poor or undifferentiated	13 (8.5)	12 (92.3)	13 (100)	4 (30.8)	6 (46.2)	8 (61.5)	13 (100)
Tumor location, n (%)*							
Colon	104 (68.0)	77 (74.0)	78 (75.0)	35 (33.7)	41 (39.4)	59 (56.7)	86 (82.7)
Rectum	30 (19.6)	24 (80.0)	28 (93.3)	8 (26.7)	12 (40.0)	21 (70.0)	29 (96.7)

CRC, colorectal cancer; *CDKN2A*, cyclin-dependent kinase inhibitor 2A; *MGMT*, O-6-methylguanine DNA methyltransferase; *MLH1*, mutL homolog 1.

*The total number of patients with CRC does not correspond because of missing data.

CRC, colorectal cancer; *CSF2*, colony stimulating factor 2; *DIS3L2*, DIS3 mitotic control homolog (*S. cerevisiae*)-like 2.

**Figure 2:**
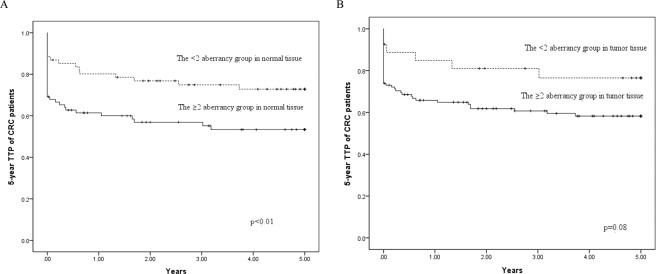
Kaplan–Meier survival curves depicting the effect of the ≥ 2 aberrancy group on 5-year TTP of CRC patients in (**A**) normal tissue and (**B**) tumor tissue. *Vertical tick marks* indicate censored events. TTP, time to progression; CRC, colorectal cancer.

We evaluated the relationship between the gene hypermethylation status and 5-year TTP, OS, and EFS of patients with CRC. In the multivariable analysis, compared with all genes in the unmethylated group, four hypermethylation genes in normal tissue were more highly associated with 5-year TTP of patients with CRC (HR 6.23; 95% CI 1.16–33.5). Moreover, a significant increasing trend of HR for 5-year TTP of patients with CRC was observed with increasing number of hypermethylation genes, both in normal tissue (*P* < 0.01) and in tumor tissue (*P* = 0.01) (Table 3). The 5-year TTP survival curves in normal tissue showed a significant difference between the group with 2 or more genes with aberrant methylation and the comparison group (*P* = 0.01). In tumor tissue, the group with two or more genes with aberrant methylation was borderline significantly associated with 5-year TTP of patients with CRC (*P* = 0.08) (Fig. 2). After multivariable adjustment for confounders, we observed a significant association between the group with two or more genes with aberrant methylation in normal tissue and 5-year TTP of patients with CRC (adjusted HR, 2.28; 95% CI, 1.17–4.44). A nonsignificant association was observed between the number of genes with hypermethylation status in both normal and tumor tissue and 5-year OS of patients with CRC (data not shown).

**Table 3:** The relationship between the number of gene hypermethylation status and 5-year TTP of CRC patients.

	Normal tissues						Tumor tissues					
	No. of subjects	No. of cases (%)	Crude		Adjusted		No. of subjects	No. of cases (%)	Crude		Adjusted	
			HR	95% CI	HR	95% CI			HR	95% CI	HR	95% CI
***NO. of hypermethylation gene***												
0	18	5 (27.8)	1.00	Referent	1.00	Referent	8	2 (25.0)	1.00	Referent	1.00	Referent
1	49	17 (34.7)	1.22	(0.39 to 3.78)	1.51	(0.33 to 6.93)	20	5 (25.0)	0.85	(0.16 to 4.64)	1.24	(0.13 to 12.0)
2	49	23 (46.9)	2.24	(0.77 to 6.52)	3.05	(0.69 to 13.5)	47	19 (40.4)	1.27	(0.28 to 5.67)	1.84	(0.23 to 14.5)
3	27	12 (44.4)	2.12	(0.66 to 6.79)	2.81	(0.57 to 13.8)	44	19 (43.2)	1.76	(0.40 to 7.66)	3.00	(0.38 to 23.6)
4	10	6 (60.0)	2.88	(0.77 to 10.8)	6.23	(1.16 to 33.5)	28	16 (57.1)	2.99	(0.69 to 13.1)	4.12	(0.53 to 32.5)
5	0	0 (0)	N/A	N/A	N/A	N/A	6	2 (33.3)	1.47	(0.21 to 10.4)	3.12	(0.26 to 36.9)
*p* for trend			0.03		<0.01				0.02		0.01	
≥2 of genes	86	41 (47.7)	1.97	(1.09 to 3.56)	2.28	(1.17 to 4.44)	125	56 (44.8)	2.03	(0.87 to 4.75)	2.34	(0.82 to 6.71)

Abbreviations: TTP, time to progression; CRC, colorectal cancer; HR, hazard ratio; CI, confidence interval; N/A, not applicable.

Adjusted for gender, age at surgery (continuous), adjuvant chemotherapy, histological grade and tumor location.

Not applicable due to limited numbers of cases.

**Figure 3:**
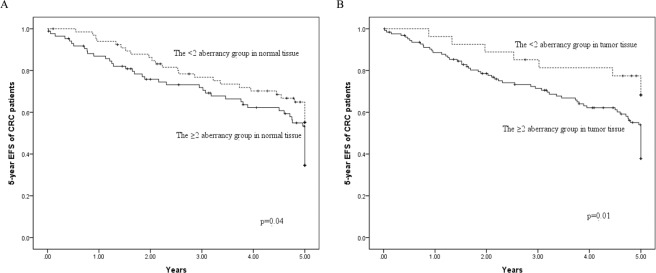
Kaplan–Meier survival curves depicting the effect of the ≥ 2 aberrancy group on 5-year EFS of CRC patients in (**A**) normal tissue and (**B**) tumor tissue. *Vertical tick marks* indicate censored events. EFS, event-free survival; CRC, colorectal cancer.

The significance of associations between four hypermethylation genes in normal tissue and 5-year EFS of patients with CRC was evaluated using Cox regression (HR, 5.85; 95% CI, 1.40–24.6). Furthermore, significant trends for increasing risk of 5-year EFS in patients with CRC were observed with increasing number of hypermethylated genes in both normal tissue (*P* < 0.01) and tumor tissue (*P* < 0.01) (Table 4). The Kaplan–Meier curves of 5-year EFS in patients with CRC among the group with two or more genes with aberrant methylation and the comparison group are shown in Fig. 3. The log-rank test revealed significant differences in both normal (*P* = 0.04) and tumor tissue (*P* = 0.01) over the entire Kaplan–Meier curve.

**Table 4:** The relationship between the number of gene hypermethylation status and 5-year EFS of CRC patients.

	Normal tissues						Tumor tissues					
	No. of subjects	No. of cases (%)	Crude		Adjusted		No. of subjects	No. of cases (%)	Crude		Adjusted	
			HR	95% CI	HR	95% CI			HR	95% CI	HR	95% CI
***NO. of gene methylation***												
0	18	5 (27.8)	1.00	Referent	1.00	Referent	8	2 (25.0)	1.00	Referent	1.00	Referent
1	49	22 (44.9)	1.51	(0.57 to 3.98)	1.69	(0.50 to 5.75)	20	6 (30.0)	1.12	(0.23 to 5.53)	1.39	(0.15 to 12.5)
2	49	26 (53.1)	191	(0.74 to 4.99)	1.70	(0.48 to 5.98)	47	23 (48.9)	2.15	(0.51 to 9.13)	2.78	(0.37 to 21.1)
3	27	16 (59.3)	2.83	(1.04 to 7.72)	3.09	(0.85 to 11.2)	44	23 (52.3)	2.42	(0.57 to 10.3)	3.60	(0.47 to 27.5)
4	10	6 (60.0)	2.72	(0.83 to 8.91)	5.85	(1.40 to 24.6)	28	19 (67.9)	4.58	(1.07 to 19.7)	6.35	(0.83 to 48.5)
5	0	0 (0)	N/A	N/A	N/A	N/A	6	2 (33.3)	1.44	(0.20 to 10.2)	2.39	(0.21 to 27.7)
*p* for trend			0.01		<0.01				<0.01		<0.01	
≥2 of genes	86	48 (55.8)	1.63	(1.01 to 2.60)	1.49	(0.87 to 2.56)	125	67 (53.6)	2.39	(1.15 to 4.99)	2.84	(1.12 to 7.22)

Abbreviations: EFS, event-free survival; CRC, colorectal cancer; HR, hazard ratio; CI, confidence interval; N/A, not applicable.

Adjusted for gender, age at surgery (continuous), adjuvant chemotherapy, histological grade and tumor location.

Not applicable due to limited numbers of cases.

We examined whether the interaction of prognosis of CRC and different cancer stages (local and advanced) was reported for the methylation status of the five genes in tumor tissues and normal tissues. Table 5 shows a significant association between the group with two or more genes with aberrant methylation in normal tissue with advanced cancer stages (Me/advanced) and 5-year TTP of patients with CRC. The crude and adjusted HRs were 9.76 (95% CI, 3.78–25.3; *P* for trend < 0.01) and 15.0 (95% CI, 3.78–25.3; *P* for trend < 0.01), respectively. We found similar results in tumor tissue, for which the crude and adjusted HRs were 6.47 (95% CI, 2.00–21.0; *P* for trend < 0.01) and 11.5 (95% CI, 1.56–85.2; *P* for trend < 0.01), respectively. As regard to the association between gene promoter region methylation and different cancer stages for 5-year OS of patients with CRC, no significant relationship was observed between *CDKN2A*, *hMLH1*, and *CSF2* methylation in the advanced cancer stages (Me/advanced) and cancer mortality in 5 years in both normal and tumor tissue (data not shown). Moreover, we estimated the association between the group with two or more genes with aberrant methylation with cancer stage and 5-year OS of patients with CRC. In normal and tumor tissue, the adjusted HRs of the group with two or more genes with aberrant methylation in advanced cancer stages (Me/advanced) were 3.97 (95% CI, 0.83–19.1; *P* for trend = 0.31) and 4.42 (95% CI, 0.57–34.5; *P* for trend = 0.10), respectively (Table 6). A significant relationship was observed between the group with two or more genes with aberrant methylation in the advanced cancer stages (Me/advanced) and 5-year EFS of patients with CRC in both normal and tumor tissue (Table 7). The group with two or more genes with aberrant methylation in the advanced cancer stages (Me/advanced) had lower 5-year EFS among patients with CRC in either normal or tumor tissue. The adjusted HRs in the group with two or more genes with aberrant methylation with advanced cancer stages (Me/advanced) were 8.04 (95% CI, 2.80–23.1; *P* for trend < 0.01) and 8.01 (95% CI, 1.92–33.4; *P* for trend < 0.01) in normal and tumor tissue, respectively.

**Table 5:** The interaction between gene promoter region methylation and different cancer stages for 5-year TTP of CRC patients.

	Normal tissues						Tumor tissues					
	No. of subjects	No. of cases (%)	Crude		Adjusted		No. of subjects	No. of cases (%)	Crude		Adjusted	
			HR	95% CI	HR	95% CI			HR	95% CI	HR	95% CI
**≧2 of genes**												
UnMe/local (1&2)	36	6 (16.7)	1.00	Referent	1.00	Referent	20	4 (20.0)	1.00	Referent	1.00	Referent
UnMe/advanced (3&4)	31	16 (51.6)	3.49	(1.21 to 10.1)	6.30	(1.39 to 28.6)	8	3 (37.5)	2.50	(0.50 to 12.4)	7.36	(0.76 to 71.7)
Me/local (1&2)	41	3 (7.3)	0.19	(0.02 to 1.60)	0.32	(0.03 to 3.74)	57	5 (8.8)	0.34	(0.07 to 1.69)	0.51	(0.04 to 5.82)
Me/advanced (3&4)	45	38 (84.4)	9.76	(3.78 to 25.3)	15.0	(3.52 to 63.6)	68	51 (75.0)	6.47	(2.00 to 21.0)	11.5	(1.56 to 85.2)
*p* for trend			<0.01		<0.01				<0.01		<0.01	

Abbreviations: TTP, time to progression; CRC, colorectal cancer; HR, hazard ratio; CI, confidence interval.

Adjusted for gender, age at surgery (continuous), adjuvant chemotherapy, histological grade and tumor location.

UnMe/loccal (1&2): gene promoter region unmethylated with cancer stage 1 or 2.

UnMe/advanced (3&4): gene promoter region unmethylated with cancer stage 3 or 4.

Me/local (1&2): gene promoter region methylated with cancer stage 1 or 2.

Me/advanced (3&4): gene promoter region methylated with cancer stage 3 or 4.

**Table 6:** The interaction between gene promoter region methylation and different cancer stages for 5-year OS of CRC patients.

	Normal tissues						Tumor tissues					
	No. of subjects	No. of cases (%)	Crude		Adjusted		No. of subjects	No. of cases (%)	Crude		Adjusted	
			HR	95% CI	HR	95% CI			HR	95% CI	HR	95% CI
≧2 of genes												
UnMe/local (1&2)	36	6 (16.7)	1.00	Referent	1.00	Referent	20	4 (20.0)	1.00	Referent	1.00	Referent
UnMe/advanced (3&4)	31	8 (25.8)	1.73	(0.60 to 5.00)	4.69	(0.98 to 22.5)	8	2 (25.0)	1.06	(0.20 to 5.81)	4.74	(0.42 to 53.2)
Me/local (1&2)	41	7 (17.1)	1.15	(0.39 to 3.41)	1.23	(0.22 to 6.91)	57	9 (15.8)	0.82	(0.25 to 2.66)	1.23	(0.14 to 10.9)
Me/advanced (3&4)	45	10 (22.2)	1.74	(0.63 to 4.79)	3.97	(0.83 to 19.1)	68	16 (23.5)	1.46	(0.49 to 4.38)	4.42	(0.57 to 34.5)
*p* for trend			0.44		0.31				0.40		0.10	

Abbreviations: OS, overall survival; CRC, colorectal cancer; HR, hazard ratio; CI, confidence interval.

Adjusted for gender, age at surgery (continuous), adjuvant chemotherapy, histological grade and tumor location.

UnMe/loccal (1&2): DNA promoter region unmethylated with cancer stage 1 or 2.

UnMe/advanced (3&4): DNA promoter region unmethylated with cancer stage 3 or 4.

Me/local (1&2): DNA promoter region methylated with cancer stage 1 or 2.

Me/advanced (3&4): DNA promoter region methylated with cancer stage 3 or 4.

**Table 7:** The interaction between gene promoter region methylation and different cancer stages for 5-year EFS of CRC patients.

	Normal tissues						Tumor tissues					
	No. of subjects	No. of cases (%)	Crude		Adjusted		No. of subjects	No. of cases (%)	Crude		Adjusted	
			HR	95% CI	HR	95% CI			HR	95% CI	HR	95% CI
**≧2 of genes**												
UnMe/local (1&2)	36	8 (22.2)	1.00	Referent	1.00	Referent	20	5 (25.0)	1.00	Referent	1.00	Referent
UnMe/advanced (3&4)	31	19 (61.3)	3.36	(1.47 to 7.68)	5.44	(1.83 to 16.1)	8	3 (37.5)	1.32	(0.32 to 5.53)	3.52	(0.58 to 21.3)
Me/local (1&2)	41	10 (24.4)	2.26	(0.50 to 3.18)	0.95	(0.26 to 3.43)	57	13 (22.8)	0.99	(0.35 to 2.77)	1.09	(0.23 to 5.25)
Me/advanced (3&4)	45	38 (84.4)	5.48	(2.55 to 11.8)	8.04	(2.80 to 23.1)	68	54 (79.4)	4.48	(1.79 to 11.2)	8.01	(1.92 to 33.4)
*p* for trend			<0.01		<0.01				<0.01		<0.01	

Abbreviations: EFS, event-free survival; CRC, colorectal cancer; HR, hazard ratio; CI, confidence interval.

Adjusted for gender, age at surgery (continuous), adjuvant chemotherapy, histological grade and tumor location.

UnMe/loccal (1&2): DNA promoter region unmethylated with cancer stage 1 or 2.

UnMe/advanced (3&4): DNA promoter region unmethylated with cancer stage 3 or 4.

Me/local (1&2): DNA promoter region methylated with cancer stage 1 or 2.

Me/advanced (3&4): DNA promoter region methylated with cancer stage 3 or 4.

## Discussion

DNA methylation, an important epigenetic mechanism, regulates gene expression through reversible modifications of histone acetylation that influence chromatin structure and the accessibility of transcription factors to their binding sites. In this study, the methylation status of five selected gene promoters (*CDKN2A*, *hMLH1*, *MGMT*, *CSF2*, and *DIS3L2*) confirmed the presence of DNA methylation in tumor tissues and matched normal tissues. We analyzed and tested the prognostic outcome, including TTP, OS, and EFS, in patients with CRC and found that the presence of hypermethylated DNA in the normal tissues could be predictive of worse outcomes in terms of TTP, OS, and EFS. Furthermore, the group with two or more genes with aberrant methylation with advanced cancer stages (Me/advanced) in normal tissue were highly associated with 5-year EFS of patients with CRC. The studies of DNA methylation biomarker panels showed that the hypermethylation of multiple candidate genes was associated with greater susceptibility to CRC^25,26^.

After correcting for potential confounders for 5-year TTP of CRC through multivariate analysis, an increasing number of hypermethylation genes was associated with poorer TTP, even in normal tissue (*P* < 0.01). In terms of OS, the group with two or more genes with aberrant methylation conferred shorter survival without reaching statistical significance. This finding is in accordance with review articles in which researchers used different panel candidate genes to predict prognosis^27,28^. Kim *et al*. evaluated the methylation status of 10 genes in patients with metastatic or recurrent CRC and found there was an independent association between a higher number of methylated genes among the genes examined and poorer clinical outcome^29^. This result is consistent with our finding that the candidate genes selected in our studies were involved in multiple molecular events in tumorigenesis. Some studies have reported no relationship between the candidate gene methylation status and prognosis in CRC^30,31^. These inconsistent results can be explained at least in part by the panels studied.

In this study, we used the other rate, EFS, which can be used to measure the prognosis in CRC clinical trials^32^. With respect to the relationship between the methylation of multiple genes and clinicopathological variables, we observed a significant relationship of normal tissue and 5-year EFS in the group with two or more genes with aberrant methylation. The result is consistent with recent findings of a trend for increases in EFS and 5-year OS for patients with gastric cancer who had zero or one methylated gene in their tumors^33^. Another study showed that hypermethylation of multiple genes was significantly associated with lower EFS in patients with neuroblastoma^34^. However, global DNA hypomethylation, which has been recognized to contribute to oncogenesis through various mechanisms, including genomic instability, resulted in the re-expression of proto-oncogenes or imprinted genes and was significantly associated with worse prognosis^35,36^.

Although genetic and environmental factors have been proposed as independent predictors of CRC prognosis^37^, the TNM staging system, which has defined the extent of cancer based solely on anatomic pathology since the 1940s, has been considered as the most comprehensive tool for predicting CRC prognosis^38,39^. Nevertheless, several studies have proposed that tumors of the same stage can differ unpredictably in both prognosis and treatment response because of heterogeneity by molecular subclassification^39,40^. This is consistent with our study of using DNA methylation patterns to perform further stratification.

Our study indicated that there was a positive association between the group with two or more genes with aberrant methylation with advanced cancer stages and prognostic outcome, including TTP and EFS, especially in normal tissue. Recently, growing evidence has demonstrated that DNA methylation profiles are changed by carcinogenic factors at the early precancerous stages in various organs^25,41–44^. Different DNA methylation profiles were observed at the chronic hepatitis or liver cirrhosis stage as a precancerous condition for liver cancer^41^. Sato *et al*. revealed aberrant DNA methylation in several genes in noncancerous tissues obtained from patients with lung cancer. The association between carcinogenetic factors such as cigarette smoking and epigenetic clustering of lung cancer based on DNA methylation profiles in adjacent lung tissue has been examined^43^. With regard to the precancerous condition for stomach cancer, aberrant DNA methylation is reportedly induced by *Helicobacter pylori* infection^44,45^. The evidence demonstrated that alterations in tissues surrounding prostate adenocarcinomas might be the result of carcinogenic factors affecting a whole organ, called a *cancer field effect*.

The surrounding tissues, which have been named *tumor indicating normal tissue* (TINT), may indicate the essence and nature of tumors. According to this information, the diagnostics and prognostics of prostate cancer could be improved^46,47^. These results were in accordance with our findings that we could find abnormalities of DNA methylation in adjacent normal tissue. The finding of aberrant DNA methylation in normal tumour-adjacent colorectal tissues could indicate a worse prognosis after surgical resection. Therefore, the TINT of CRC could be an alternative source of prognostic markers to help in clinical decision making.

The most frequently studied biomarker was *CDKN2A* (*p16*)^48^, which is as a negative regulator in the G_1_/S phase of the cell cycle by disrupting the complexes of CDK4^42^. Studies including subgroup evaluations have demonstrated that aberrant *CDKN2A* methylation was significantly correlated to a poor prognosis^49,50^, even though the finding of Sanz-Casla *et al*. demonstrated an inconsistent result^51^. The DNA mismatch-repair system is inactivated by *MLH1* hypermethylation, which reduces the MLH1 protein expression and stops the formation of *MLH1* protein and blocks the activation of mismatch repair (MMR) genes. Inactivation of DNA mismatch repair caused by promoter hypermethylation of *MLH1* lead to cell proliferation and genomic instability to the point of CRC formation^52^. The significant finding by Iida *et al*. and Kuan *et al*. showed that *MLH1* hypermethylation was associated with worse prognosis in TNM stages 1 to 4^23,53^.

*MGMT* is in charge of repairing DNA damage produced by alkylating agents. Aberrant *MGMT* methylation may be involved in CRC tumorigenesis^54^. For *MGMT* promoter hypermethylation, Kuan *et al*. showed that *MGMT* methylation in TNM stages 3 to 4 indicates a poor prognosis. However, the study of Nilsson *et al*. showed conflicting results with a better prognosis^28^.

With regard to *CSF2*, Lee *et al*. clarified that *CSF2* was the mainly upregulated gene of importance for carcinoma development and invasiveness among those involved in positive regulation of tyrosine phosphorylation of STAT5. *CSF2* could play an as an important role of prognosticator and a future therapeutic target of urothelial carcinoma^55^. *DIS3L2* inactivation has been connected to modified expression of mitotic checkpoint proteins and mitotic abnormalities. Knockdown of *DIS3L2* enhanced the growth of human cancer cells, and overexpression prohibited these cells growth^21^. There are few studies assessed the relationship between *CSF2* or *DIS3L2* promoter methylation and CRC prognosis

The findings of the this study must be interpreted within the context of some limitations. An important consideration in assessing the relation between the five selected gene promoters methylation and the CRC prognostic outcome due to *KRAS* and *BRAF* mutation analysis was not conducted in this study. There is mutually exclusiveness between KRAS and BRAF, worse survival in patients with methylated *p16* and *BRAF* mutations is not influenced by *KRAS* status^23,27^. Furthermore, the present study did not include subjects with colorectal benign adenoma and healthy individuals. The development of an acceptable protocol could help in the study of the methylation status of tumor suppressor genes; their distribution in promoter regions; their distribution in the proximal colon, distal colon, and rectum; and their time sequence dependence in healthy individuals, particularly in those who develop CRC. Wu *et al*. used an animal model to simulate the methylation status of the adenoma–carcinoma sequence, which is a precursor of animal cancer progression; but they did not study humans^18^. Finally, the results of the present study should be carefully interpreted because of the small number of patients who were analyzed. A larger prospective cohort study is warranted to validate these results.

In summary, this study demonstrated that DNA methylation status was significantly associated with poor CRC prognosis, particularly in the matched normal tissues with advanced stage, because the molecular changes could not be examined on the basis of clinical pathology. To mark out the implication of DNA aberrant methylation in CRC scenario, future research addressing the relationship should be prospective and make attempts to include subjects with colorectal benign adenoma and healthy subjects. We suggest using these findings in the matched normal tissues of patients with CRC as an alternative source of prognostic markers to assist in clinical decision making.
