# Author Correction: Multiple gene promoter methylation and clinical stage in adjacent normal tissues: Effect on prognosis of colorectal cancer in Taiwan

**DOI:** 10.1038/s41598-020-60646-7

**Published:** 2020-02-21

**Authors:** Chih-Hsiung Hsu, Cheng-Wen Hsiao, Chien-An Sun, Wen-Chih Wu, Tsan Yang, Je-Ming Hu, Yu-Chan Liao, Chi-Hua Huang, Chao-Yang Chen, Fu-Huang Lin, Yu-Ching Chou

**Affiliations:** 1 Graduate Institute of Medical Sciences, National Defense Medical Center, Taipei, Taiwan, Republic of China; 2 Teaching Office, Tri-Service General Hospital, National Defense Medical Center, Taipei, Taiwan, Republic of China; 3 Division of Colorectal Surgery, Department of Surgery, Tri-Service General Hospital, National Defense Medical Center, Taipei, Taiwan, Republic of China; 4 Department of Public Health, College of Medicine, Fu Jen Catholic University, New Taipei City, Taiwan, Republic of China; 5 Big Data Research Center, College of Medicine, Fu Jen Catholic University, New Taipei City, Taiwan, Republic of China; 6 School of Public Health, National Defense Medical Center, Taipei, Taiwan, Republic of China; 7 Department of Surgery, Suao and Yuanshan branches of Taipei Veterans General Hospital, Yilan County, Taiwan, Republic of China; 8 Department of Health Business Administration, Meiho University, Pingtung County, Taiwan, Republic of China; 9 Adjunct Instructor, School of Medicine, National Defense Medical Center, Taipei, Taiwan, Republic of China

Correction to: *Scientific Reports* 10.1038/s41598-019-56691-6, published online 10 January 2020

This Article contains errors in Table 2 where the first instance of “CSF2” should read “CDKN2A”, the first instance of “DIS3L2” should read “hMLH1” and the first instance of “≥2 of genes” should read “MGMT.”

In addition, this Article contains errors in reference 2, which was incorrectly given as:

“Ferlay, J. et al. (2017).”

The correct reference is listed below as ref. 1.
